# Influence of *Lactobacillus* Biosurfactants on Skin Permeation of Hydrocortisone

**DOI:** 10.3390/pharmaceutics13060820

**Published:** 2021-05-31

**Authors:** Angela Abruzzo, Carola Parolin, Elisa Corazza, Barbara Giordani, Massimiliano Pio di Cagno, Teresa Cerchiara, Federica Bigucci, Beatrice Vitali, Barbara Luppi

**Affiliations:** 1 Department of Pharmacy and Biotechnology, Alma Mater Studiorum, University of Bologna, Via San Donato 19/2, 40127 Bologna, Italy; carola.parolin@unibo.it (C.P.); elisa.corazza7@unibo.it (E.C.); barbara.giordani4@unibo.it (B.G.); teresa.cerchiara2@unibo.it (T.C.); federica.bigucci@unibo.it (F.B.); b.vitali@unibo.it (B.V.); barbara.luppi@unibo.it (B.L.); 2 Department of Pharmacy, Faculty of Mathematics and Natural Sciences, University of Oslo, Sem Sælands vei 3, 0371 Oslo, Norway; m.p.d.cagno@farmasi.uio.no

**Keywords:** biosurfactants, *Lactobacillus*, hydrocortisone, drug solubility, skin permeation

## Abstract

One of the most widely used strategies to improve drug diffusion through the skin is the use of permeation enhancers. The aim of this work was to investigate the effect of two biosurfactants (BS), produced by *Lactobacillus crispatus* BC1 and *Lactobacillus gasseri* BC9, on the skin permeation profile of hydrocortisone (HC, model drug). HC aqueous solubility and in vitro diffusion studies through porcine skin were performed in the presence of BC1-BS and BC9-BS at concentrations below and above critical micellar concentrations (CMC). Moreover, skin hydration tests and differential scanning calorimetry (DSC) analysis were performed to further investigate BS interaction with the outermost layer of the skin. Both BS increased HC solubility, especially at concentrations above their CMC. At concentrations below the CMC, drug permeation through the skin was improved, as the result of a dual effect: a) the formation of a superficial lipophilic environment, as confirmed by the reduction in skin hydration and b) the interaction between BS and the stratum corneum (SC), as demonstrated by the DSC curves. From the obtained data, it appears that BC1-BS and BC9-BS could represent new promising green excipients for drug permeation enhancement through the skin.

## 1. Introduction

The growing interest in transdermal drug delivery may be ascribed to advantageous features such as easy accessibility, non-invasiveness, prevention from hepatic first-pass metabolism, minimal toxic side effects, and high patient compliance [1,2]. However, efficient permeation of therapeutic compounds through the skin is still challenging because of its anatomy, which has evolved to impede the flux of toxins into the body and to minimize water loss. As a result, the skin naturally represents a barrier for the penetration of foreign molecules. The outermost layer of the skin, known as the stratum corneum (SC), is mainly responsible for this barrier function and consists of a lipid-rich matrix with embedded nonviable, anucleate, and keratinized cells (keratinocytes). Specifically, lipid molecules in the SC, e.g., ceramides, cholesterol, and fatty acids, play a major role in controlling the permeation of chemical and biological drugs [3].

Various strategies have emerged to optimize transdermal delivery, and these can be categorized into physical and non-physical approaches. The active approach involves physical or mechanical methods (e.g., using ultrasonic waves, electroporation, or microneedles), whereas the passive one entails the use of formulations enriching the skin with substances able to modify the chemical composition of the diffusion barrier [2,4]. A broad range of chemical agents have been tested to promote drug absorption through the skin, including surfactants, which have gained great importance [1,5,6]. As amphiphilic molecules, surfactants are able to increase both the water solubility and the skin permeability of drugs depending on the concentration at which they are used. In fact, below the critical micellar concentration (CMC), surfactants are present as single molecules and mainly promote drug permeation by disrupting the structure and packing of lipids and proteins within the SC. Conversely, above the CMC, surfactants assemble into micelles, whose core represents a suitable environment in which lipophilic drugs can be efficiently solubilized [7]. Chemical surfactants do not derive from sustainable resources and are reported to produce relevant side effects in vivo, including allergic reactions and skin irritations [8]. The exact mechanisms responsible for these effects are still unclear, but they are probably linked to the physiochemical properties of surfactants, to the molecule’s concentration, and to their permanence on the epidermis [8]. Consequently, there is a growing need for alternative compounds with improved properties in terms of biocompatibility [9,10]. 

Biosurfactants (BS) share their amphiphilic molecular structure with chemical surfactants but are produced by microorganisms as secondary metabolites, and their components (sugars, lipids, and proteins) are similar to those present in skin cells’ membrane (phospholipid and proteins) [9,11,12]. Major attention was given to BS produced by generally recognized as safe (GRAS) microbes. Along with their biocompatibility, BS show other numerous appealing properties that make them suitable for the design of innovative drug delivery systems such as microemulsions, nanoparticles, and liposomes [13,14,15,16]. These natural surfactants exert significant surface and emulsifying properties together with stability at extreme conditions of pH, temperature, and salinity [9,17]. 

Recently, two different BS isolated from human *L. crispatus* BC1 and *L. gasseri* BC9 (BC1-BS and BC9-BS) were characterized and evaluated for their possible application as promising antimicrobial agents. Specifically, BC1-BS and BC9-BS showed their ability to interfere with the adhesion and biofilm of *Candida* [18], with biofilms of methicillin-resistant *Staphylococcus aureus* [16], and they have been proposed as green pharmaceutical excipients for the development of new vesicular carriers containing conventional antibiotics [15,19]. Both BS are composed by peptide-like molecules containing different fatty acid carbon chains. BC1-BS and BC9-BS are able to reduce the surface tension, and they show CMC values equal to 2.5 mg/mL and 2.0 mg/mL, respectively. In this study, we investigated their possible application as permeation enhancers for transdermal delivery of a model drug, i.e., hydrocortisone (HC). Particularly, the ability of BC1-BS and BC9-BS to improve HC aqueous solubilization and skin permeation was evaluated. Moreover, BS’ effect on skin hydration and BS’ interaction with the SC were investigated in order to suggest possible mechanisms favoring HC skin permeation. 

## 2. Materials and Methods

### 2.1. Materials

Tween 80 was provided from Fluka (Milan, Italy). Hydrocortisone and all chemicals and solvents (HPLC grade ≥ 98%) were purchased from Sigma-Aldrich (Milan, Italy). Buffer solution at pH 7.4 was composed of 7.4 mM Na_2_HPO_4_×12 H_2_O, 1.1 mM KH_2_PO_4_, and 136 mM NaCl. The culture media and GasPak EZ were supplied by Becton Dickinson and Company (Sparks, MD, USA). l-cysteine hydrochloride monohydrate was purchased from Merck (Darmstadt, Germany). 

### 2.2. Production and Isolation of L. crispatus BC1 and L. gasseri BC9 Biosurfactants 

The isolation of cell-bound biosurfactants from *L. crispatus* BC1 and *L. gasseri* BC9 (BC1-BS and BC9-BS) was conducted following a procedure previously published by our research group [15,16,18]. Briefly, 100 mL of an overnight lactobacilli culture was inoculated in 900 mL of de Man, Rogosa and Sharpe (MRS) broth and allowed to grow anaerobically for 24 h. Cell pellets were harvested by centrifugation (10,000× *g*, 15 min), washed twice in sterile water, and re-suspended in 300 mL of PBS. The suspensions were gently stirred at room temperature for 2 h to release the cell-bound BS. The supernatants were isolated by centrifugation, filtered through a 0.22 μm pore size filter (PES 0.22 μm syringe filters, VWR International, Milan, Italy), purified by dialysis against demineralized water in a Cellu-Sep© membrane (molecular weight cutoff 6000–8000 Da; Spectra/Por 2 dialysis membrane Spectrum Laboratories Inc., Rancho Dominguez, CA, USA) for 24 h at room temperature, and freeze dried at 0.01 atm and −45 °C (Christ Freeze Dryer ALPHA 1–2, Milan, Italy). 

### 2.3. Surface-Activity and Critical Micelle Concentration of BC1-BS and BC9-BS

The concentration at which surfactant molecules aggregate and form micelles in an aqueous environment (CMC) was measured by the ring method using a tensiometer (K8600E Krüss GmbH, Hamburg, Germany) equipped with a 1.9 cm platinum ring, as previously described [15]. The surface tension (dyne/cm) of saline solutions (NaCl 0.9% *w*/*v*) with different biosurfactant concentrations (0.016–8.0 mg/mL for BC1-BS and 0.004–5.65 mg/mL for BC9-BS) was measured at room temperature. The surface tension of Tween 80 in saline solution was also determined (0.003–0.5 mg/mL). CMC was determined by plotting the surface tension as a function of the BS concentration logarithm and is represented as the point at which the baseline of the minimal surface tension intersects the slope where surface tension shows a linear decline.

### 2.4. HC Solubility 

Excess HC was dispersed in a saline solution under agitation for 48 h at 25 °C. In order to remove the undissolved HC, the dispersion was centrifuged at 5890× *g* for 15 min, and afterwards, the supernatant was collected and filtered through a 0.22 µm pore-size cellulose acetate syringe filter (VWR International, Milan, Italy). The obtained supernatant was immediately assayed for HC content by high-performance liquid chromatography (HPLC). 

### 2.5. HPLC Analytical Assay 

The analytical assay was conducted following the procedure previously reported in [20] with some modifications by using a Shimadzu (Milan, Italy) LC-10ATVP chromatographic pump and a Shimadzu SPD-10AVP UV–vis detector set at 244 nm. Separation was obtained on a Phenomenex (Torrance, California) Sinergy Fusion-RP 80A (150 × 4.6 mm^2^ i.d., 5 µm) coupled to a Phenomenex Security Guard C18 guard cartridge (4 × 3.0 mm^2^ i.d., 5 µm). For samples containing BC9-BS and BC1-BS, the mobile phase was composed of a mixture of acetonitrile/ethanol/phosphate buffer at pH 7.4 50:10:40 (*v*/*v*) and a mixture of acetonitrile/phosphate buffer at pH 7.4 30:70 (*v*/*v*), respectively. The flow rate was 0.4 mL/min and manual injections were made using a Rheodyne 7125 injector with a 20 µL sample loop. Data processing was carried out by means of a CromatoPlus computerized integration system (Shimadzu Italia, Milan, Italy). Different calibration curves in ethanol/saline solution (9:1, *v*/*v*) and in PBS and ethanol (8:2 *v*/*v*) were obtained with drug concentrations ranging from 5.43 µg/mL to 108.60 µg/mL and from 0.50 µg/mL to 6.0 µg/mL, respectively. For both curves, a good linearity was observed (R^2^ = 0.999). Limits of detection (LOD) and quantification (LOQ) were 0.15 µg/mL and 0.50 µg/mL, respectively. The methodology granted good precision, with RSD values always lower than 5.0%.

### 2.6. Influence of BC1-BS and BC9-BS on HC Solubility

The influence of BC1-BS and BC9-BS on HC aqueous solubility was determined in the presence of biosurfactant concentrations up to 10-fold their respective CMC. In particular, an excess drug amount was dispersed in saline solution at 25 °C containing BC1-BS (1.25–25 mg/mL) and BC9-BS (1.00–20 mg/mL). Tween 80 (0.025–0.1 mg/mL) was selected as non-ionic surfactant, generally considered to be less irritating than ionic ones [21] and was tested as a control. After 48 h, undissolved HC was removed by centrifugation and filtration (conditions as above) and the drug concentration was determined by HPLC.

### 2.7. Tissue Preparation

Porcine ear skin was selected as model because of its similarity to the human skin regarding morphology and permeation characteristics [22,23]. Freshly excised pig ear was obtained from a local abattoir (CLAI, Faenza, Italy) and washed with saline solution. Skin was carefully separated from cartilage using a scalpel within 3 h of the animal’s death. Following excision and removal of subcutaneous tissue, the full-thickness skin was stored frozen at −20 °C on aluminum foil. The integrity of the full-thickness skin sample was assessed measuring the electrical resistance (voltage: 100 mV, frequency: 100 Hz; Agilent 4263B LCR Meter, Microlease, I). The specimen was used for a permeation experiment if the value was equal to or above 1.57 kΩ/cm^2^, in agreement with electrical resistance cut-off values reported by Davies et al. [24]. 

### 2.8. Effect of BS on Skin Hydration

As previously reported, skin hydration is a sensitive parameter which reflects the effects of excipients on the skin [10,25]. For this study, 50 μL of solutions containing Tween 80, BC1-BS, and BC9-BS at a concentration below and above (1/2-fold lower and 10-fold higher) their respective CMC were applied on full-thickness porcine skin (area equal to 1.77 cm^2^). Skin hydration was evaluated after 60 min at 20 ± 1 °C and 40–50% relative humidity through a Corneometer^®^ CM 825 (Courage+Khazaka electronic GmbH, Köln, Germany). The hydration of porcine skin treated with saline solution was measured as control.

### 2.9. Influence of BC1-BS and BC9-BS on HC Permeation

In vitro permeation studies were performed to evaluate the effect of different concentrations (below and above their CMC) of surfactants (Tween 80, BC1-BS, and BC9-BS) on HC permeation through pig ear skin. A Franz-type static glass diffusion cell (15 mm jacketed cell with a flat-ground joint and clear glass with a 12 mL receptor volume; diffusion surface area = 1.77 cm^2^) equipped with a V6A Stirrer (PermeGearInc., Hellertown, PA, USA) was used. The control sample and surfactant-containing samples, prepared as described in Section 2.4 and Section 2.6, respectively, were tested. 

The receptor medium was composed of 12 mL of PBS and ethanol (80:20 *v*/*v*), maintained at 32 ± 1 °C by means of a surrounding jacket and constantly stirred to assure a uniform drug concentration. The full-thickness skin was clamped between the donor and receptor compartments with the SC facing upwards and the dermal side in contact with the receptor medium, ensuring that no air bubbles were below the skin. At the starting point of the experiment, 200 μL of each sample were applied on the skin in the donor compartment. At predetermined time intervals (1 h) until 7 h, aliquots of 200 μL of receptor medium were removed from the sampling port followed by replacement with an equal volume of fresh receptor solution and were analyzed by HPLC. Sink conditions were assured during the test.

Cumulative amounts of drug permeated per unit area of pig skin (µg/cm^2^) were plotted against time (minutes). The flux (*j*) was calculated from the slope of the linear section of the curve (from approximately 60 to 420 min [20]). Taking into account the starting drug concentration (*c*_0_) within different samples, the permeability coefficient (*Kp*) was calculated according to the following equation [26]:

(1) $$Kp=\frac{j}{c_{0}}$$

The enhancement ratio (*ER*) was used to evaluate the effect of the surfactant on the HC permeation, and it was calculated according to the following equation [27]:

(2) $$ER=\frac{Kp\;in\;the\;presence\;of\;surfactant}{Kp\;without\;surfactant}$$

### 2.10. Differential Scanning Calorimetry Analysis (DSC) of Stratum Corneum

DSC analysis was used to evaluate the interaction between BC1-BS and BC9-BS and the main components of the SC [28,29]. Firstly, the epidermis was isolated after immersing the full-thickness porcine skin in water at 60 °C for 2 min and then peeling it off with forceps. Subsequently, to prepare SC sheets, isolated epidermis was soaked in 1% (*w*/*v*) trypsin in pH 7.4 PBS at 4 °C for 15 h. The epidermis was removed with a cotton swab, and obtained SC sheets were individually washed 3 times with distilled water. The isolated SC sheets were cut in pieces with the same weight (25 mg) and put in contact with 3 mL of BS saline solutions at a concentration 1/2-fold lower than their CMC for 7 h. Then, the SC sheets were rinsed with water to remove the excess of BC1-BS, BC9-BS, and salts and were dried in a desiccator containing CaCl_2_ for 48 h. The control SC sample was prepared with the same procedure without BC1-BS and BC9-BS. DSC analysis was performed using a Perkin Elmer DSC 6 (Perkin Elmer, Beaconsfield, UK) with nitrogen as a purge gas (20 mL/min). Samples, weighing 2–3 mg, were placed in an aluminum pan and heated from 25 to 160 °C at a scanning rate of 10 °C/min.

### 2.11. Statistical Analysis

All results are shown as mean ± standard deviation (SD). SD was calculated from the values of 3 independent experiments, except for the permeation results, which were calculated from the values of at least 5 independent experiments. Data from all experiments were analyzed using a *t*-test, and differences were deemed significant for *p* < 0.05. 

## 3. Results and Discussion

BC1-BS and BC9-BS, produced by *L. crispatus* BC1 and *L. gasseri* BC9, respectively, were previously characterized in terms of chemical and functional properties [15,18]. Both BS presented a peptide-like moiety linked to fatty acid carbon chains. Specifically, BC1-BS contained an amino acid portion attributable to tyrosine, serine, proline, glycine, and arginine linked to fatty acids, such as β-hydroxytridecanoic acid (3-OH-C13), β-hydroxytetradecanoic acid (3-OH-C14), β-hydroxypentadecanoic acid (3-OH-C15), and β-hydroxyhexadecanoic acid (3-OH-C16) [18]. BC9-BS was composed by amino acids, such as histidine, valine, and threonine and fatty acids with higher chain lengths (C14-C17) [15]. 

### 3.1. Surface-Activity Determination and Critical Micelle Concentration of Biosurfactants

**Figure 1:**
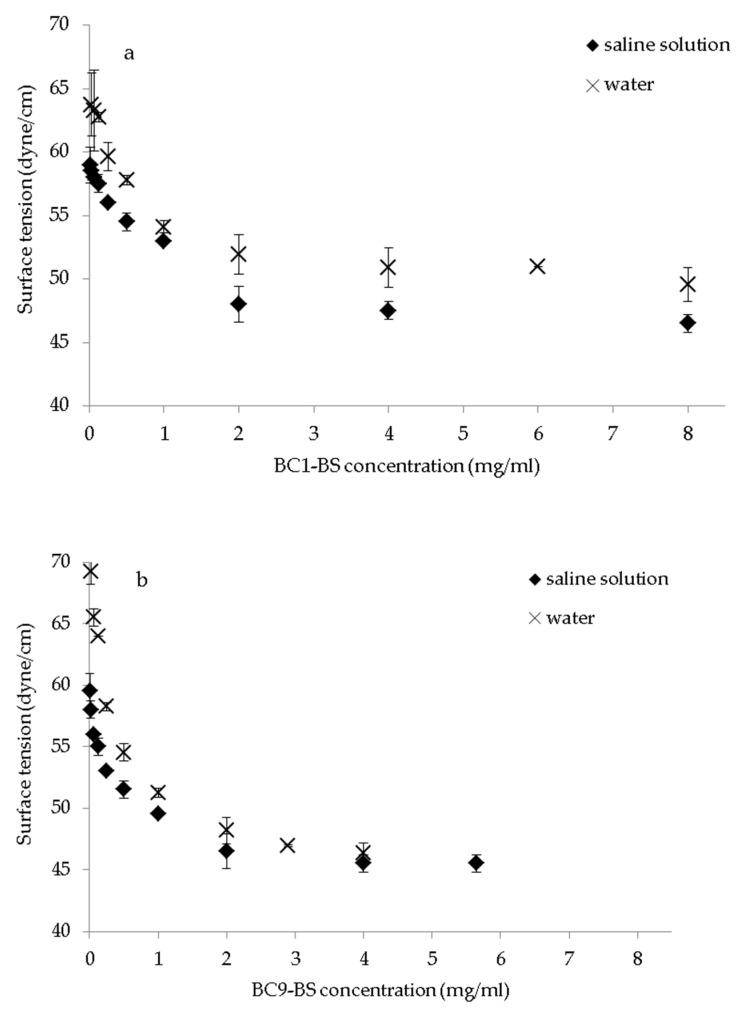

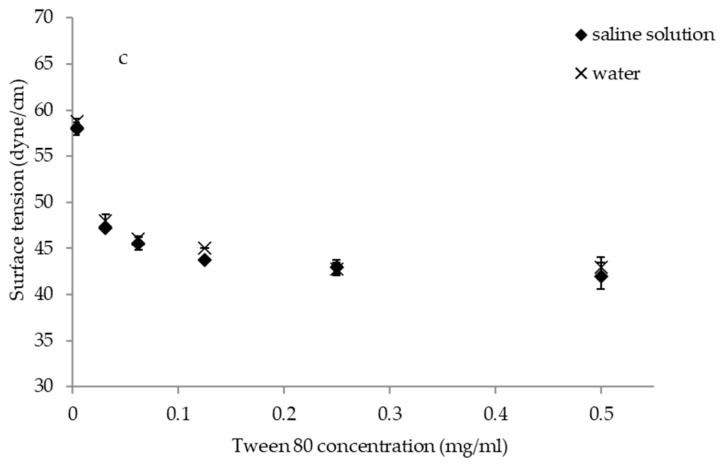
Surface tension values versus (**a**) BC1-BS, (**b**) BC9-BS, and (**c**) Tween 80 concentrations (mg/mL). Data are plotted as mean values of surface tension (dyne/cm) ± SD (*n* = 3).

Our previous studies demonstrated that the surface tension of BC1-BS and BC9-BS in water decreased as biosurfactant concentration increased [15,18]. In this study, the surface-activity, and the CMC of biosurfactants were also determined in a saline solution. Figure 1a,b shows the comparison between surface tension values obtained by dissolving BC1-BS and BC9-BS in water or in a saline solution. When BC1-BS biosurfactant was solubilized at concentrations from 0.016 mg/mL to 8.0 mg/mL in saline, the surface tension decreased from 59.0 ± 1.4 to 46.5 ± 0.7 dyne/cm, whereas, in the case of BC9-BS at concentrations ranging from 0.004 mg/mL to 5.65 mg/mL, the surface tension decreased from 59.5 ± 0.7 to 45.5 ± 0.7 dyne/cm. Overall, for both BS, a reduction in surface tension values was observed in the saline solution with respect to water (*p* < 0.05). This result could be attributed to the presence of positively charged amino acids: arginine for BC1-BS [18] and histidine for BC9-BS [15]. As reported in the literature, for ionic surfactants, the presence of electrolytes causes a decrease in surface tension as a result of a lower repulsion between similarly charged ionic head groups [30]. However, the CMC values determined for BC1-BS and BC9-BS in saline solution did not differ from the values obtained in water (*p* > 0.05); they were 2.5 mg/mL and 2.0 mg/mL, respectively. On the contrary, the surface-activity of Tween 80 was not influenced by the presence of sodium chloride in water (Figure 1c). This result was in agreement with previous findings confirming that, in the case of non-ionic surfactants, the repulsion between the head groups is not a limiting factor for micellization and that no change in CMC upon addition of salt is observed [31].

### 3.2. HC Solubility 

HC solubility in saline solution was detected by HPLC analysis of the clear solution obtained after centrifugation and subsequent filtration of the drug suspension. The solubility of the drug in saline solution at 25 °C was 0.197 ± 0.015 mg/mL. This value was lower to that obtained in water (0.295 ± 0.003 mg/mL; [20]) because of the salting out effect [32,33].

### 3.3. Influence of BC1-BS and BC9-BS on HC Solubility

The measurement of drug solubility in a defined vehicle is a crucial factor in the development of a drug delivery system to avoid drug crystallization and precipitation. This parameter is also useful for an appropriate evaluation of drug permeation across the skin. In fact, the drug concentration gradient and the partition coefficient between the vehicle and the skin are affected by drug solubility in the vehicle itself [34].

**Figure 2:**
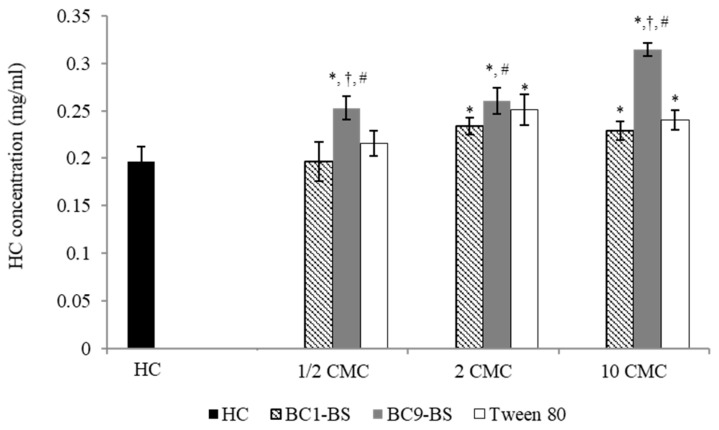
Solubility of HC at 25 °C as a function of surfactant concentration below (1/2-fold lower that their CMC) and above (2-fold and 10-fold higher than their CMC) the CMC. Data are expressed as means ± SD, *n* = 3. Significance indicated by * = *p* < 0.05 compared to HC solubility without surfactants, by # = *p* < 0.05 compared to HC solubility in the presence of same concentration of BC1-BS, and by † = *p* < 0.05 compared to the HC solubility in the presence of the same concentration of Tween 80.

Figure 2 reports the influence of the tested surfactants (BC1-BS, BC9-BS, and Tween 80) at different concentrations (below and above their CMC) on drug solubility. 

At concentrations below the CMC (1/2-fold lower than CMC), no significant difference with respect to the control (*p* > 0.05) was observed in the presence of BC1-BS and Tween 80, whereas a slight increase in drug solubility was obtained with BC9-BS (*p* < 0.05), indicating BC9-BS’s ability to interact with the drug as single molecules. 

At concentrations above the CMC (2-fold and 10-fold higher than their CMC), for all the tested surfactants, an increase in HC solubility with respect to the control (*p* < 0.05) was observed, as a consequence of the drug’s entrapment into micelles. Moreover, at a concentration 10-fold higher than its CMC, BC9-BS allowed the dissolution of the highest amount of HC (*p* < 0.05), and HC solubility increased from 0.197 ± 0.015 mg/mL to 0.314 ± 0.007 mg/mL. Additionally, BC9-BS allowed us to obtain a slight increase in HC solubility with respect to BC1-BS at a concentration 2-fold higher than its CMC (*p* < 0.05). These results were probably related to the particular composition of BC9-BS that was characterized by the presence of fatty acids with a longer hydrocarbon chain with respect to BC1-BS. In fact, as reported in the literature, the solubilization capacity of the micelles increased significantly with the increase in the hydrophobic chain length of the surfactant [35,36,37]. Moreover, the peptide portion of BC9-BS, composed of histidine, valine, and threonine, could contribute to the improvement of drug solubility. This behavior was in agreement with previous findings [38], in which a greater ability of histidine to increase ofloxacin solubility with respect to other amino acids (including serine, phenylalanine, isoleucine, methionine, and tyrosine) was reported.

### 3.4. Effect of BS on Skin Hydration

**Figure 3:**
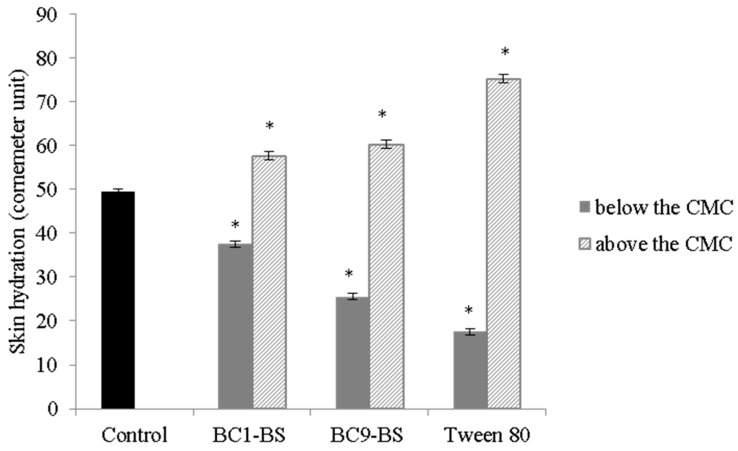
Skin hydration values (Corneometer unit) after treatment with surfactants at a concentration below and above the CMC. Data are expressed as means ± SD, *n* = 3. Significance indicated by * = *p* < 0.05 compared to the control.

In order to evaluate surfactant influence on skin properties, skin hydration was measured in the presence of the tested surfactants. Figure 3 shows the values of skin hydration (Corneometer) after 60 min of treatment with BC1-BS, BC9-BS, and Tween 80 at concentrations below (1/2-fold lower than their CMC) and above the CMC (10-fold higher than their CMC). For all the tested surfactants at concentrations below the CMC, a reduction in skin hydration values with respect to the control was observed (*p* < 0.05). This behavior was probably attributed to the presence of the fatty acid portions of surfactants that could form a lipophilic environment on the skin surface. Our conclusion was in agreement with data obtained in the work of Knoth and co-workers, in which the authors observed the same trend with a biosurfactant obtained from the corn wet-milling industry [10] sharing the same lipopeptide nature of BC1-BS and BC9-BS. Moreover, previous findings reported that lipid nanoparticles, able to provide a lipid film on top of the skin, led to a decrease in the measured skin hydration [39,40,41]. Interestingly, BC9-BS led to a greater reduction in the hydration skin with respect to BC1-BS, probably as a consequence of the presence of fatty acids with a longer hydrocarbon chain. 

On the other side, in the presence of surfactant concentration above the CMC, a greater skin hydration was observed with respect to the control. This result could be attributed to the formation of micelles that determined the assembling of the hydrophobic parts of surfactants and consequently limited the formation of the lipophilic film on the skin surface. Moreover, the exposure of the outer hydrophilic parts of micelles could contribute to increase the water retention on the skin surface [42]. Furthermore, no significant difference between skin hydration in the presence of BC1-BS and BC9-BS at concentrations above the CMC was observed (*p* > 0.05). 

### 3.5. Influence of BC1-BS and BC9-BS on HC Permeation

**Figure 4:**
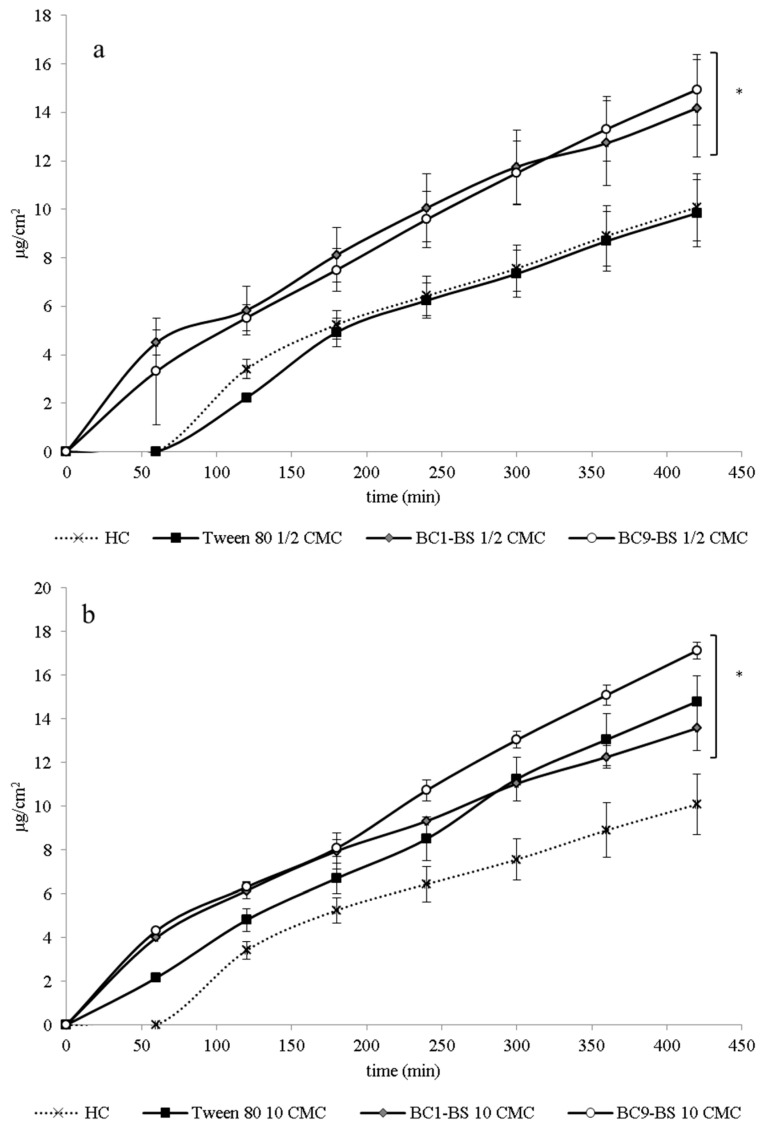
Permeation profiles through porcine skin of HC in the absence and presence of different concentrations of surfactant: (**a**) concentrations below CMC (1/2-fold lower than CMC); (**b**) concentrations above CMC (10-fold higher than CMC). Data are expressed as means ± SD, *n* = 5. Significance indicated by * = *p* < 0.05 compared to the control.

Figure 4 reports the cumulative amount of HC permeated through porcine full-thickness skin plotted against time in the presence of BC1-BS, BC9-BS, and Tween 80 at concentrations below (1/2-fold lower than CMC) and above the CMC (10-fold higher than CMC). All permeability profiles showed good linearity from approximately the first 1 h until 7 h. No lag time was observed for the tested samples with the exception of the control and the Tween-80-containing sample, thus demonstrating an increase in drug diffusibility.

After 7 h, the HC amount permeated in the control sample was equal to 10.08 ± 1.39 µg/cm^2^. 

At a concentration 1/2-fold lower than the CMC, no significant difference (*p* > 0.05) was observed between drug permeation in the presence of Tween 80 (9.84 ± 1.38 µg/cm^2^; j = 1.20 ± 0.23 µg/cm^2^ h) and the control (10.09 ± 1.39 µg/cm^2^; j = 1.17 ± 0.20 µg/cm^2^ h). Instead, a significant increase in drug permeation was obtained when BS-based samples were applied on the skin (*p* < 0.05 vs. control). The amount of HC permeated through the skin was equal to 14.18 ± 2.01 µg/cm^2^ (j = 1.50 ± 0.20 µg/cm^2^ h) and 14.94 ± 1.44 µg/cm^2^ (j = 1.91 ± 0.17 µg/cm^2^ h) in the presence of BC1-BS and BC9-BS, respectively. This behavior could probably be explained by the BS’ ability to interact with the main components of the skin, leading to an environmental change in the SC and, consequently, increasing drug partition and diffusion through the skin. This conclusion was also confirmed by the DSC results (see Section 3.6) and is in agreement with previous findings reporting good penetration-enhancing properties for biosurfactant molecules with amphiphilic and lipophilic natures together with a high content of fatty acids [10]. Additionally, the formation of a more lipophilic skin surface, as demonstrated by the reduction in skin hydration (Section 3.4), could also contribute to the improvement in skin HC permeation [10,43,44]. 

At concentrations above the CMC, for all the tested surfactants, an increase in HC permeation with respect to the control (*p* < 0.05) was observed as a consequence of a drug solubility increase (see Section 3.3). Interestingly, at a concentration 10-fold higher than its CMC, BC9-BS allowed the highest HC permeation (17.13 ± 0.39 µg/cm^2^; j = 2.21 ± 0.05 µg/cm^2^ h) among all the samples (*p* < 0.05). As reported in Section 3.3, the presence of BC9-BS at a concentration 10-fold higher than its CMC allowed us to obtain the highest drug solubility value among all the samples. In this condition, the presence of the micelles favored an increase in water retention on the skin surface (see Section 3.4) that could limit drug partition and diffusivity. However, the increase in drug solubility in the same condition could lead to an improved concentration gradient and thus passive diffusion through the skin, in agreement with other studies [45,46,47,48]. 

**Figure 5:**
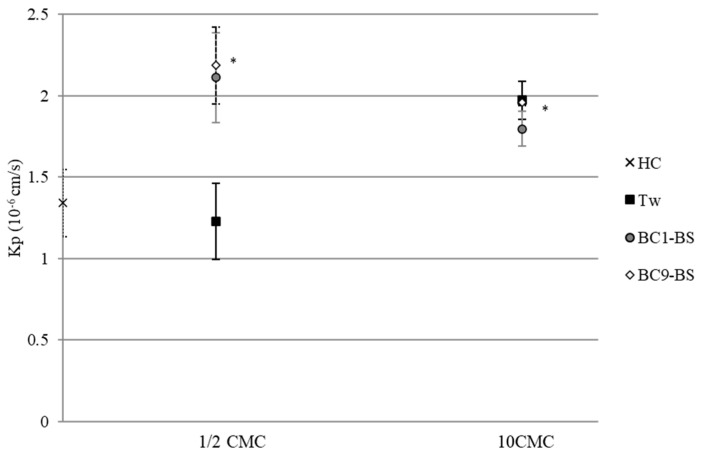
HC permeability coefficients (Kp) in the absence and presence of various surfactant concentrations. Data are expressed as means ± SD, *n* = 5. Significance indicated by * = *p* < 0.05 compared to Kp of HC.

Considering that each selected BS concentration provided the solubilization of different amounts of drug, the normalization of fluxes to the initial drug concentration *c*_0_ was useful to compare the different samples. Figure 5 reports the permeability coefficients Kp (cm/s) for the tested samples calculated through Equation (1). In the presence of Tween 80 at a concentration below its CMC, no significant difference was observed with respect to the control (*p* > 0.05). Interestingly, in the presence of both BS at a concentration below the CMC, a greater Kp was obtained with respect to the control and Tween 80 (*p* < 0.05). Again, this result was probably due to a dual effect: (i) the biosurfactant’s ability to interact with the skin as single molecules, thus providing a positive environmental change and consequently favoring drug permeation; (ii) the presence of the lipophilic surface environment, which was able to promote HC partition and diffusion through the skin (increased solubilization of HC). 

Moreover, despite being well known that micelles do not penetrate the skin because of steric hindrance [49], for all the tested BS, at a concentration 10-fold higher than CMC, HC permeability coefficients were higher than the Kp of the control (*p* < 0.05). 

Finally, at concentrations below the CMC, the ER obtained in the presence of BC1-BS and BC9-BS were equal to 1.63 and 1.57, respectively, and higher than the ER value obtained for Tween 80 (0.92), demonstrating the superior ability of BS to enhance drug skin absorption with respect to the chemical surfactant. The ER values obtained in the presence of BS were similar to those obtained in our previous work, in which surfactants were obtained from itaconic acid and tested in similar conditions [20]. Another study, reporting experimental conditions similar to those used in this study, is the work of Sarheed [50]. In this study, the authors determined the ER in the presence of SDS at different concentrations (0.25 and 1%); the values obtained with SDS were higher than the ER measured for BC1-BS and BC9-BS. Despite these results, we concluded that BS could represent a valid alternative to SDS, considering the high toxicity of the latter [20]. 

### 3.6. Differential Scanning Calorimetry Analysis (DSC) of Stratum Corneum

In order to elucidate the mechanism by which BS favored drug permeation, differential scanning calorimetry analysis (DSC) of the SC was performed. DSC is one of the most useful technique to gain information on penetration enhancer ability to affect the order degree of the lipid bilayers in the skin [51]. It has been proposed that the DSC profile of porcine SC presents different characteristic endothermic peaks near 65 °C, 75 °C, and 105 °C because of the thermal transitions involving intercellular lipids, lipid–protein complexes, and intercellular keratin, respectively [52,53]. Generally, the shift in the peak at 65 and 75 °C to lower temperatures was intended as the disruption of the lipid bilayer [54,55]. 

**Figure 6:**
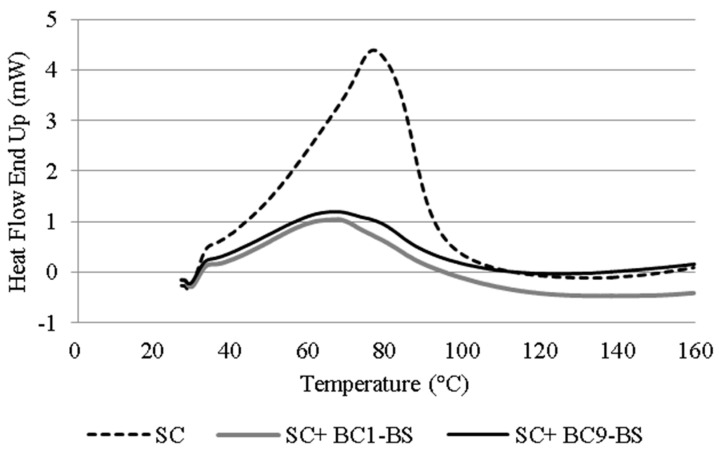
DSC curves of the SC obtained from porcine skin treated with BC1-BS and BC9-BS at a concentration below their CMC.

In our study, the DSC thermogram of the SC not treated with the enhancer showed a clear endotherm peak at 77 °C (Figure 6). The thermogram of the SC incubated with BC1-BS and BC9-BS showed a shift in this endothermic peak to lower temperatures (at 68 and 67 °C for BC1-BS and BC9-BS, respectively). This change suggested that the incorporation of BS into the SC at a concentration below the CMC resulted in the disruption and/or fluidization of the lipid bilayer and thus the idea that HC diffusion through the skin could be improved by this mechanism. 

## 4. Conclusions

To the best of our knowledge, this study is the first that investigates the use of molecules derived from human *Lactobacillus* strains as transdermal permeation enhancers. Particularly, BS isolated from *L. crispatus* BC1 and *L. gasseri* BC9 were shown to exert a dual positive effect on HC, which was used as a model drug. Firstly, BS led to an increase in drug solubility. Secondly, BS increased HC permeation through the porcine skin. The latter result was due to both the formation of a superficial lipophilic environment and the interaction of BS molecules with the SC. In conclusion, these molecules could be proposed as alternative skin permeation enhancers to chemical compounds. 

Future investigation will be focused on the evaluation of BS’ ability to improve the permeation of drugs with different physico-chemical properties.

## Data Availability

Data is contained within the article.
